# MHY1485 ameliorates UV-induced skin cell damages via activating mTOR-Nrf2 signaling

**DOI:** 10.18632/oncotarget.14299

**Published:** 2017-01-04

**Authors:** Bo Yang, Qiu-Yun Xu, Chun-Yan Guo, Jin-Wen Huang, Shu-Mei Wang, Yong-Mei Li, Ying Tu, Li He, Zhi-Gang Bi, Chao Ji, Bo Cheng

**Affiliations:** ^1^ Department of Dermatology, Longhua Hospital, Shanghai University of Traditional Chinese Medicine, Shanghai, China; ^2^ Department of Dermatology, The First Affiliated Hospital of Fujian Medical University, Fuzhou, China; ^3^ Department of Dermatology, The First Affiliated Hospital of Kunming Medical University, Yunnan Provincial Institute of Dermatology, Kunming, China; ^4^ Department of Dermatology, BenQ Medical Center, Nanjing Medical University, Nanjing, China

**Keywords:** ultra violet (UV), skin cell damage, MHY1485, mTOR, Nrf2

## Abstract

Ultra Violet (UV)-caused skin cell damage is a main cause of skin cancer. Here, we studied the activity of MHY1485, a mTOR activator, in UV-treated skin cells. In primary human skin keratinocytes, HaCaT keratinocytes and human skin fibroblasts, MHY1485 ameliorated UV-induced cell death and apoptosis. mTOR activation is required for MHY1485-induced above cytoprotective actions. mTOR kinase inhibitors (OSI-027, AZD-8055 and AZD-2014) or mTOR shRNA knockdown almost abolished MHY1485-induced cytoprotection. Further, MHY1485 treatment in skin cells activated mTOR downstream NF-E2-related factor 2 (Nrf2) signaling, causing Nrf2 Ser-40 phosphorylation, stabilization/upregulation and nuclear translocation, as well as mRNA expression of Nrf2-dictated genes. Contrarily, Nrf2 knockdown or S40T mutation almost nullified MHY1485-induced cytoprotection. MHY1485 suppressed UV-induced reactive oxygen species production and DNA single strand breaks in skin keratinocytes and fibroblasts. Together, we conclude that MHY1485 inhibits UV-induced skin cell damages via activating mTOR-Nrf2 signaling.

## INTRODUCTION

Ultra Violet (UV) radiation in skin keratinocytes and fibroblasts would lead to oxidative stress and DNA damages, along with activation of several signal transduction pathways that are important for cancer initiation and progression [1–3]. Our group [4–9] has been dedicated to understand the underlying mechanisms of UV-induced skin cell damages, and to develop possible anti-UV strategies.

mammalian target of rapamycin (mTOR) is a vital pro-survival signaling [10]. There are two functionally distinct multi-protein mTOR complexes, namely the mTOR complex 1 (mTORC1) and the mTOR complex 2 (mTORC2) [10]. mTORC1 shall be inhibited by rapamycin or its analogs, and is formed with mTOR, Raptor, mLST8 and several others [10–12]. mTORC1 phosphorylates p70S6K1 (S6K1) and eukaryotic-translation initiation factor 4E-binding protein 1 (4E-BP1) to promote protein translation, energy metabolism and cell survival [11, 13]. On the other hand, the rapamycin-insensitive mTORC2 is compose of mTOR, Rictor and Sin1 [10–12]. The complex serves as the upstream kinase of Akt (Ser-473), a major pro-survival signaling [11, 13]. It has been increasingly clear that both complexes are important for cell survival [10, 12].

Choi *et al*., recently developed a cell-permeable, small-molecule mTOR specific activator, named MHY1485 [14]. This compound has been shown to directly bind to mTOR, and to activate mTOR at μM concentrations [14]. MHY1485 could induce phosphorylation of mTOR (at Ser-2448) to significantly increase its activity [14, 15]. In the current study, we show that MHY1485 inhibits UV-induced skin cell damages via activating mTOR signaling.

## RESULTS

### MHY1485 inhibits UV-induced skin cell death

**Figure 1:**
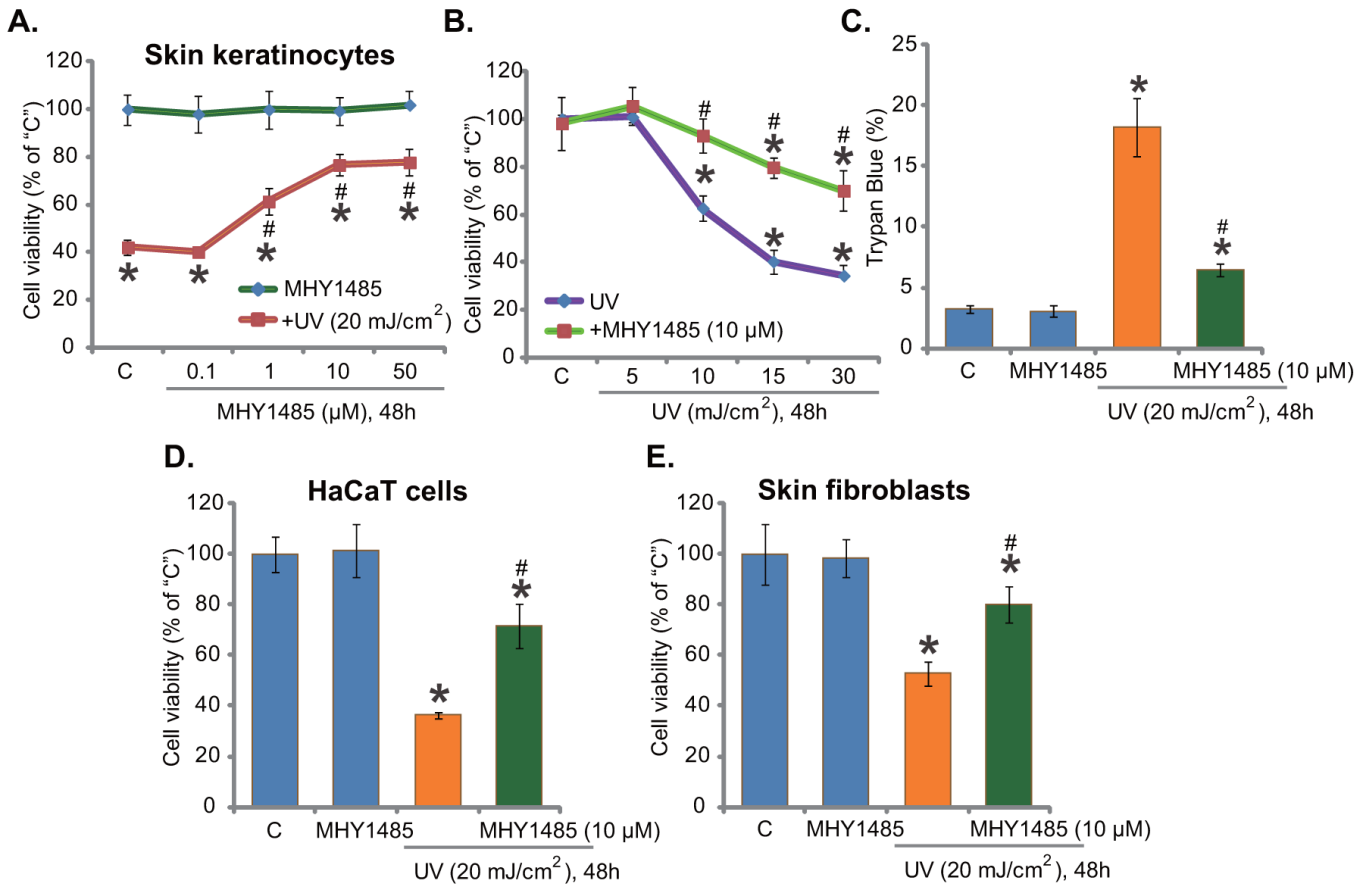
MHY1485 inhibits UV-induced skin cell death Human skin keratinocytes **A-C**. HaCaT keratinocytes **D**. or human skin fibroblasts **E**. pre-treated for 30 min with designated concentration of MHY1485 (1-50μM), were irradiated with UV at applied intensity (5-30 mJ/cm^2^), cells were further cultured in the complete medium for 48 hours; Cell survival was tested by MTT assay (A, B, E and F); Cell death was tested by Trypan blue staining assay (C). The values were expressed as mean ± standard deviation (SD) (Same for the following figures). All experiments were repeated three times and similar results were obtained (Same for the following figures). “C” stands for medium-treated control group (Same for the following figures). * *P* < 0.05 *vs*. “C” group. ^#^ *P* < 0.05 *vs*. UV irradiation only group.

Here, we aim to understand the potential effect of MHY1485 on UV. Primary cultured human skin keratinocytes [7] were irradiated with UV (20 mJ/cm^2^), MTT assay results in Figure 1A showed that cell survival (MTT OD) was decreased sharply (over 50%) following UV radiation. Remarkably, pre-treatment with MHY1485 (1-50 μM) significantly attenuated UV-induced viability reduction (Figure 1A). MHY1485 displayed a dose-dependent response in protecting skin keratinocytes from UV (Figure 1A). At a very low concentration (0.1 μM), MHY1485 failed to inhibit UV damages (Figure 1A). MHY1485 alone, at tested concentrations (1-50 μM), failed to change cell survival (Figure 1A). Since 10 μM MHY1485 displayed superior efficiency in protecting skin keratinocytes from UV (Figure 1A), this concentration was selected for future mechanistic studies.

Skin keratinocytes were also irradiated with UV at other intensities (5-30 mJ/cm^2^), MHY1485 (10 μM) pre-treatment was again cytoprotective under these UV doses (Figure 1B). Results of the trypan blue staining assay showed that UV (20 mJ/cm^2^)-induced death of skin keratinocytes was ameliorated with pre-treatment of MHY1485 (10 μM) (Figure 1C). The potential effect of MHY1485 on UV radiation in other skin cells was also examined. As displayed, in HaCaT keratinocytes (Figure 1D) and primary human skin fibroblasts (Figure 1E), MHY1485 (10 μM) remarkably inhibited UV (20 mJ/cm^2^)-induced viability reduction. Together, these results demonstrate that MHY1485 pre-treatment inhibits UV-induced skin cell death.

### MHY1485 attenuates UV-induced skin cell apoptosis

**Figure 2:**
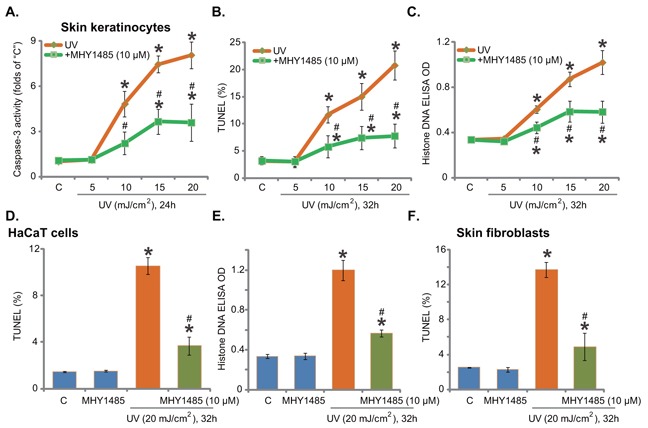
MHY1485 attenuates UV-induced skin cell apoptosis Human skin keratinocytes **A-C**. HaCaT keratinocytes **D-E**. or human skin fibroblasts **F**. pre-treated for 30 min with MHY1485 (10 μM), were irradiated with UV at applied intensity (5-20 mJ/cm^2^), cells were further cultured in the complete medium for applied time; Cell apoptosis was tested by the listed assays. * *P* < 0.05 *vs*. “C” group. ^#^ *P* < 0.05 *vs*. UV irradiation only group.

Next, using the methods described previously [7], we tested the potential effect of MHY1485 on UV-induced skin cell apoptosis. In line with our previous findings [7], UV radiation, at 10-20 mJ/cm^2^, increased caspase-3 activity (Figure 2A), TUNEL-positive nuclei (Figure 2B) and Histone DNA ELISA optic density (OD) value (Figure 2C) in skin keratinocytes, indicating profound apoptosis activation. Significantly, pre-treatment with MHY1485 (10 μM) largely attenuated UV-provoked apoptosis in skin keratinocytes (Figure 2A–2C). UV radiation at 5 mJ/cm^2^ was unable to induce significant apoptosis activation (Figure 2A–2C). The similar anti-apoptosis activity by MHY1485 was also observed in UV-irradiated HaCaT keratinocytes (Figure 2D and 2E) and primary skin fibroblasts (Figure 2F). MHY1485 (10 μM) alone didn't induce apoptosis in the skin cells (Figure 2A–2F).

### Activation of mTOR is required for MHY1485-induced cytoprotection against UV

**Figure 3:**
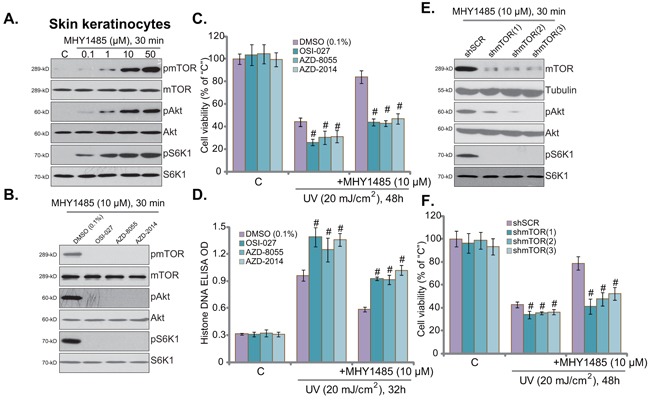
Activation of mTOR is required for MHY1485-induced cytoprotection against UV Skin keratinocytes were treated with MHY1485 (0.1-50 μM) for 30 min, expression of mTOR-associated proteins was tested **A**. Skin keratinocytes were treated with MHY1485 (10 μM) for 30 min, or plus mTOR kinase inhibitor (OSI-027, AZD2014 or AZD-8055, 200 nM each), expression of listed proteins was shown **B**. Human skin keratinocytes, pre-treated for 30 min with MHY1485 (10 μM) or plus indicated the mTOR kinase inhibitor, were irradiated with UV (20 mJ/cm^2^), cells were further cultured in the complete medium for applied time; Cell viability **C**. (MTT assay) and apoptosis **D**. (Histone DNA ELISA assay) were tested. Puromycin-selected stable skin keratinocytes, expressing indicated mTOR shRNA (“shmTOR1/2/3”) or scramble control shRNA (“shSCR”), were pre-treated for 30 min with MHY1485 (10 μM), followed by UV (20 mJ/cm^2^) radiation, cells were further cultured in the complete medium for 48 hours; Cell survival was tested (F). Expression of listed proteins in above cells with MHY1485 (10 μM, 30 min) treatment was shown **E**. ^#^ *P* < 0.05 *vs*. DMSO (0.1%) group (**C** and **D**) or “shSCR” group **F**.

MHY1485 is a novel small-molecular mTOR activator [14, 16]. Western blot assay results in Figure 3A demonstrated that MHY1485 treatment in skin keratinocytes indeed activated mTOR, which was evidenced by phosphorylation (“p-”) of mTOR (Ser-2448), S6K1 (Thr-389), and Akt (Ser-473) (Figure 3A). As discussed, p-S6K1 was the indicator of mTORC1 activation, and p-Akt at Ser-473 reflected mTORC2 activation [10–12]. MHY1485 displayed dose-dependent response in activating mTOR (Figure 3A). Notably, to exclude the influence of medium serum on mTOR activation, cells were starved with warm PBS before MHY1485 treatment [17].

To study the link between mTOR activation and MHY1485-induced skin cell protection, various mTOR kinase inhibitors were applied, including OSI-027, AZD-8055 and AZD-2014 [18, 19]. Expectably, these mTOR inhibitors almost completely blocked MHY1485-induced mTOR activation (p-mTOR/S6K1/Akt Ser473) in skin keratinocytes (Figure 3B). Remarkably, MHY1485-induced cytoprotection against UV was almost nullified in the presence of above mTOR inhibitors (Figure 3C and 3D). Intriguingly, these mTOR blockers also aggravated UV-induce skin keratinocyte cell death (Figure 3C) and apoptosis (Figure 3D), indicating the function of basal mTOR activation in promoting cell survival against UV.

The above pharmacological evidences suggest that mTOR activation is required for MHY1485-induced cell protection against UV. To further support this hypothesis, shRNA strategy was applied. As described, a total of three different mTOR shRNAs (“shmTOR1/2/3”) targeting non-overlapping sequence of mTOR were utilized. Each of the applied mTOR shRNA led to dramatic mTOR downregulation in skin keratinocytes (Figure 3E). Consequently, MHY1485-induced mTOR activation was almost blocked by mTOR shRNAs (Figure 3E). Consequently, MHY1485-induced cytoprotection against UV was largely compromised in the mTOR-silenced keratinocytes (Figure 3F). In another words, MHY1485 failed to protect skin keratinocytes when mTOR was silenced (Figure 3F). These results provided genetic evidence to show that mTOR activation is required for MHY1485-induced cytoprotection against UV. Again, skin keratinocytes with mTOR shRNA were more sensitive to UV damages (Figure 3F), further support the cytoprotective effect of mTOR against UV radiation. The above pharmacological and genetic experiments were repeated in human skin fibroblasts, and similar results were obtained (Data not shown).

### MHY1485 activates mTOR downstream Nrf2 signaling to protect skin keratinocytes from UV

**Figure 4:**
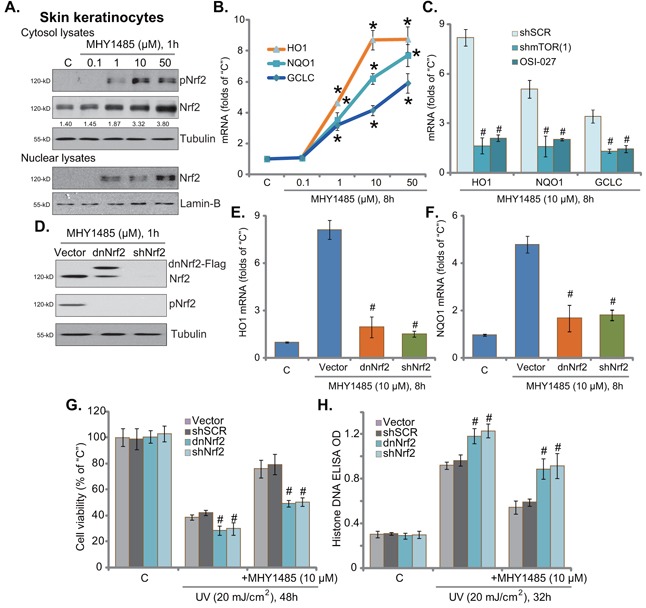
MHY1485 activates mTOR downstream Nrf2 signaling to protect skin keratinocytes from UV Skin keratinocytes were treated with MHY1485 (0 1-50 μM) for applied time, expression of listed proteins in cytosol and nuclei was shown **A**. mRNA expression of listed Nrf-2 genes was tested by RT-qPCR assay **B**. Puromycin-selected stable skin keratinocytes, expressing mTOR shRNA (“shmTOR1”) or scramble control shRNA (“shSCR”), were treated with MHY1485 (10 μM) or plus OSI-027 (200 nM), cells were further cultured for 8 hours, expression of listed mRNAs was tested **C**. Puromycin-selected stable skin keratinocytes, expressing scramble control shRNA (“shSCR”), Nrf2 shRNA (“shNrf2”), dominant negative Nrf2 (S40T, “dnNrf2”, Flag-tagged) or empty vector (“Vector”, pSV2 puro-Flag), were treated with MHY1485 (10 μM), cells were further cultured in complete medium for applied time; Expression of listed proteins was shown **D**. Relative *HO1* **E**. and *NQO1* **F**. mRNA expression was tested; Above cells were also subjected to UV (20 mJ/cm^2^) radiation, or plus MHY1485 (10 μM, 30 min prior UV), cell viability **G**. and apoptosis **H**. were tested. * *P* < 0.05 *vs*. “C” group (B). ^#^ *P* < 0.05 *vs*. “shSCR” group (C). ^#^ *P* < 0.05 *vs*. “Vector” group (E-H).

Recent studies have suggested that mTOR could activate its potential downstream NF-E2-related factor 2 (Nrf2), a key anti-oxidant signaling [20–22], to inhibit oxidative stress and promote cell survival [23, 24]. We therefore analyzed Nrf2 signaling in MHY1485-treated skin keratinocytes. Western blot assay results demonstrated that MHY1485 dose-dependently induced Nrf2 phosphorylation (at Ser-40) and cytosol accumulation in skin keratinocytes (Figure 4A). Further, Nrf2 nuclear translocation was also observed following MHY1485 treatment (Figure 4A). Consequently, mRNA expressions of Nrf2-dictated genes, including *heme oxygenase-1 (HO1)*, *NAD(P)H quinone oxidoreductase 1 (NQO1)* and *γ-glutamyl cystine ligase catalytic subunit (GCLC)* [21], were significantly increased with MHY1485 (1-50 μM) treatment (Figure 4B). Importantly, MHY1485 (10 μM)-induced transcription of above genes was largely inhibited by the mTOR kinase inhibitor OSI-027 or mTOR shRNA (Figure 4C). These results suggest that MHY1485 activated mTOR downstream Nrf2 signaling in skin keratinocytes.

To study the function of Nrf2 activation in MHY1485-induced cytoprotection, we again utilized genetic strategies from our previous study [7] to interfere Nrf2 activation. As demonstrated, Nrf2 shRNA knockdown (Figure 4D) or S40T mutation (Figure 4D) almost abolished MHY1485 (10 μM)-induced mRNA expression of *HO1* (Figure 4E)*, NQO1* (Figure 4F) and *GCLC* (Data not shown). More intriguingly, Nrf2 silence or mutation in skin keratinocytes almost abolished MHY1485-induced cytoprotection against UV (Figure 4G and 4H). MHY1485 was largely in-effective against UV when Nrf2 was silenced or mutated (Figure 4G and 4H). These results imply that Nrf2 Ser40 phosphorylation and activation, as the downstream of mTOR, is required for MHY1485-induced cytoprotection. In line with our previous results [7], skin keratinocytes with Nrf2 silence or mutation were more vulnerable to UV (Figure 4G and 4H), once again confirming the cytoprotective effect of mTOR in skin cells. The above experiments were also repeated in human skin fibroblasts, and similar results were obtained (Data not shown).

### MHY1485 attenuates UV-induced ROS production and DNA damages

**Figure 5:**
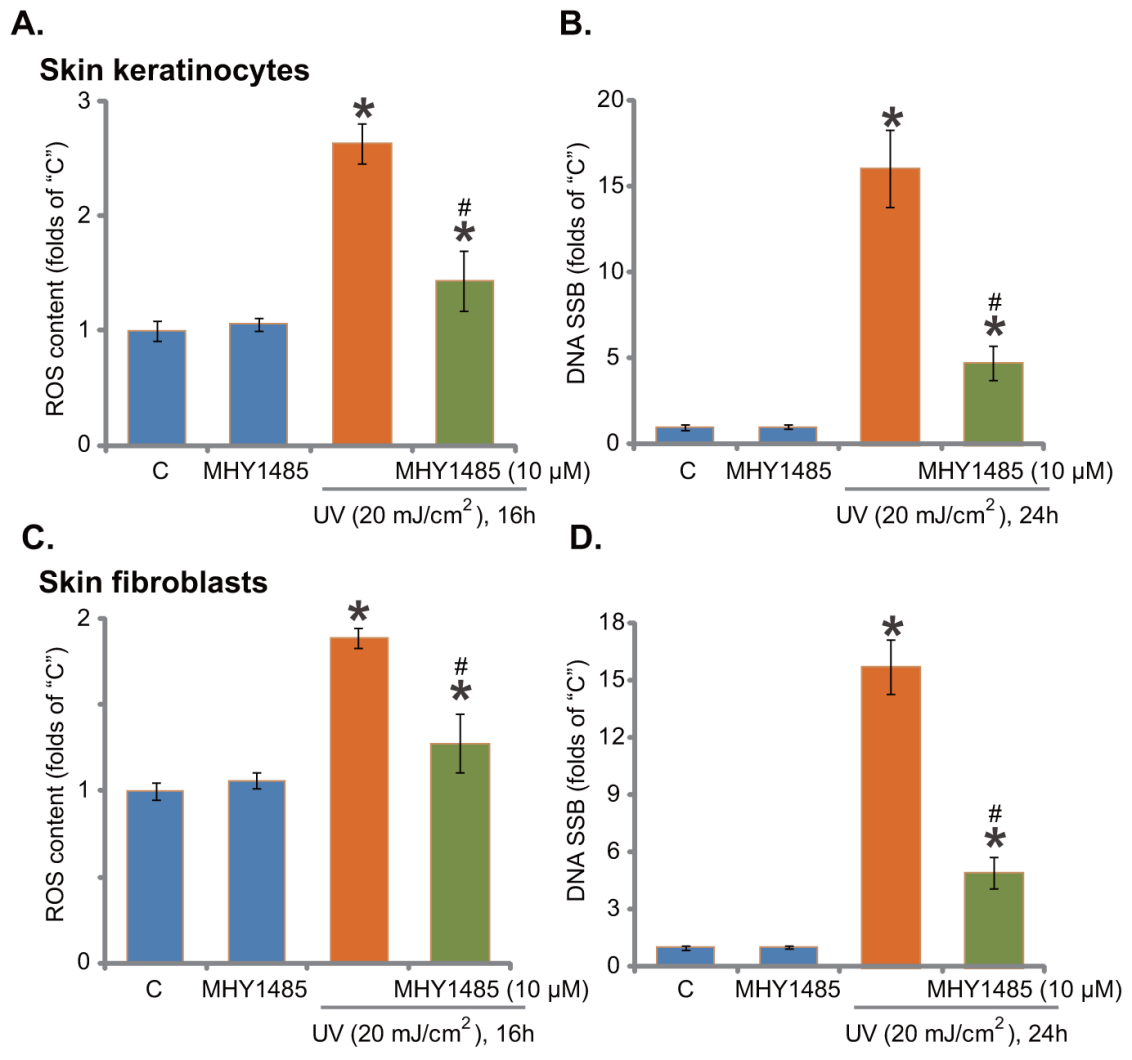
MHY1485 attenuates UV-induced ROS production and DNA damages Skin keratinocytes **A**. and **B**. or skin fibroblasts **C**. and **D**. pre-treated for 30 min with MHY1485 (10 μM), were irradiated with UV (20 mJ/cm^2^), cells were further cultured in the complete medium for applied time; Relative ROS production and DNA single strand breaks (SSB) were tested by the described assays * *P* < 0.05 *vs*. “C” group. ^#^ ***P*** < 0.05 *vs*. UV irradiation only group.

Growth evidences have indicated that activation of Nrf2 signaling could inhibit UV-induced reactive oxygen species (ROS) production and DNA damages [24–26]. Our recent study demonstrated that gremlin activated Nrf2 and inhibited UV-induced ROS production and subsequent DNA single strand break (SSB) [7]. Since MHY1485 activated Nrf2 signaling, its potential anti-oxidant activity was analyzed next. As demonstrated, pre-treatment with MHY1485 (10 μM, 30 min) indeed dramatically attenuated UV (20 mJ/cm^2^)-induced ROS production in skin keratinocytes (Figure 5A). As a result, UV-induced DNA SSB was largely attenuated (Figure 5B). The similar results were also observed in the skin fibroblasts, where MHY1485 (10 μM) decreased UV-induced oxidative stress (Figure 5C) and DNA damages (Figure 5D). MHY1485 (10 μM) alone, as expected, didn't change ROS content and SSB level (Figure 5A–5D). Collectively, these results demonstrate that MHY1485 attenuates UV-induced ROS production and DNA damages in skin cells.

## DISCUSSION

Here, we found that MHY1485 activated mTOR and significantly attenuated UV-induced death and apoptosis of skin keratinocytes, HaCaT keratinocytes and skin fibroblasts. Activation of mTOR is required for MHY1485-induced above actions. mTOR inhibitors (OSI-027, AZD-8055 and AZD-2014) or mTOR shRNAs almost completely abolished MHY1485-exerted cytoprotection against UV.

As one of the uppermost anti-oxidant signalings in mammalian cells, Nrf2 dictates transcription of multiple key anti-oxidant genes to protect cells from oxidative stress [22, 27–29]. Intriguingly, recent studies have suggested that mTOR could also function as a potential upstream signaling for Nrf2 [23, 24, 26]. For instance, Zhang *et al*., demonstrated that Nrf2 activation by Salvianolic acid requires mTOR activation [23]. Salvianolic acid A induced mTOR-dependent Nrf2 phosphorylation (at Ser-40) and accumulation [23]. Li *et al*., demonstrated that 3H-1,2-dithiole-3-thione (D3T) activated Nrf2 via phosphorylation at Ser-40 in a mTOR-dependent manner [24]. Our previous study also showed that gremlin activated Akt-mTOR and Nrf2 signaling, then protected skin cells from UV [7].

In the current study, we provided compelling evidences to support that MHY1485 activated Nrf2 signaling in skin cells. MHY1485 induced Nrf2 phosphorylation at Ser-40, which might cause it departure from its suppressor KEAP1 and subsequent stabilization [23, 24, 30, 31]. Indeed, Nrf2 expression was increased in MHY1485-treated cells. Further, Nrf2 nuclear localization was noticed following MHY1485 treatment in skin keratinocytes, which presumably led to transcription of several Nrf2 genes (*HO1, NQO1*, *GCLC*). Nrf2 S40T mutation or shRNA knockdown almost abolished above gene expression by MHY1485. Importantly, activation of Nrf2 is important for MHY1485-induced actions in skin keratinocytes. Nrf2 knockdown or mutation almost abolished MHY1485-induced cytoprotection against UV. Thus, we propose that mTOR downstream Nrf2 activation mediates MHY1485-induced skin cell protection against UV.

Groups including ours [4, 7, 32] have been focusing on the development of the agents that may inhibit or even reverse UV-induced DNA damages, which might discontinue the transformation process [33–36]. Here, we found that MHY1485 significantly inhibited UV-induced ROS production and following DNA damages in skin keratinocytes and fibroblasts. Thus, this novel mTOR activator might be further tested as a promising strategy for skin cancer prevention.

## MATERIALS AND METHODS

### Chemicals and reagents

MHY1485 andmTOR kinase inhibitors OSI-027, AZD-8055 and AZD-2014 were obtained from MCE China (Shanghai, China). All antibodies of this study were obtained from Cell Signaling Technology (Nanjing, China). The cell culture regents were purchased from Gibco (Suzhou, China).

### Cell culture and UV radiation

The culture of the primary human skin keratinocytes, HaCaT keratinocytes and human skin fibroblasts were described in detail in our previous studies [4–7]. UV radiation procedures were also described previously [4, 8, 9].

### Cell survival and cell death assays

MTT cell viability assay and cell death trypan blue staining assay were described in our previous studies [4–6].

### Cell apoptosis assay

Following treatment of cells, apoptosis was tested by Histone DNA apoptosis ELISA assay,TUNEL (Terminal deoxynucleotidyl transferase dUTP nick end labeling) staining assay, or the caspase-3 activity assay. The detailed protocols of these assays were described previously [4–7].

### Real-time quantitative PCR (“RT-qPCR”) analysis

RNA extraction and RT-qPCR were depicted in our previous studies [7]. All the primers of the Nrf2 genes were provided by Dr. Jiang [24, 26].

### Western blot assay

Western blot assay was depicted previously [4–6]. For detection of nuclear proteins, the cell nuclei were isolated by the nuclei isolation kit purchased from Sigma [23]. Indicated protein band (in total gray) was quantified via the ImageJ software [37].

### Reactive oxygen species (ROS) detection

Following treatment of cells, the fluorescent dye dihydrorhodamine (DHR) was applied to test cellular ROS content via the FACS machine (Beckton Dickinson FACScan, Suzhou, China). The ROS fluorescent intensity of treatment group was normalized to that of untreated control group [4, 7].

### Measure of DNA single strand breaks (SSB)

The detailed protocol for analyzing DNA SSB was described previously [4, 7]. SSB intensity in UV-irradiated cells was always normalized to the control level.

### mTOR shRNA knockdown

The three different mTOR shRNAs (“shmTOR1/2/3”) with non-overlapping sequence were produced by constructing the GV248 vector (Genepharm, Shanghai, China) with a puromycin resistance gene and targeted shRNA. Stable knockdown by shRNA was described in detail previously [4–6]. Briefly, skin cells were cultured with 50-60% confluence. mTOR shRNA or the scramble control shRNA (“shSCR”, Santa Cruz Biotech) was added to the cells for 24 hours. Cells were then selected by puromycin (5.0 μg/mL) for 4-5 days. Afterwards, knockdown of mTOR was confirmed by Western blot assay.

### Nrf2 knockdown and mutation

Nrf2 shRNA knockdown, S40T dominant negative mutation, and the stable cell selection were described in detail in our previous study [7].

### Statistical analysis

All data were normalized to control values of each assay and were presented as mean ± standard deviation (SD). Data were analyzed by one-way ANOVA with SPSS 16.0 software (SPSS Inc., Chicago, IL). Significance was chosen as *P*< 0.05.
